# Evaluation of colorectal liver metastases using virtual monoenergetic images obtained from dual-layer spectral computed tomography

**DOI:** 10.1007/s00261-024-04635-8

**Published:** 2024-10-15

**Authors:** Jae Seok Bae, Jeong Hee Yoon, Jae Hyun Kim, Seungchul Han, Sungeun Park, Se Woo Kim

**Affiliations:** 1 Department of Radiology, Seoul National University Hospital, Seoul, Republic of Korea; 2 Department of Radiology, Seoul National University College of Medicine, Seoul, Republic of Korea; 3 Department of Radiology, Samsung Medical Center, Seoul, Republic of Korea; 4 Department of Radiology, Konkuk University Medical Center, Seoul, Republic of Korea; 5 Department of Radiology, Konkuk University School of Medicine, Seoul, Republic of Korea

**Keywords:** Colorectal liver metastasis, Virtual monoenergetic imaging, Dual-layer spectral computed tomography

## Abstract

**Purpose:**

To assess the potential of virtual monoenergetic images in assessing colorectal liver metastasis (CRLM) compared with conventional CT images.

**Methods:**

This single-center, retrospective study included 173 consecutive patients (mean age, 65.5 ± 10.6 years; 106 men) who underwent dual-layer spectral CT (DLSCT) between November 2016 and April 2021. Portal venous phase images were reconstructed using hybrid iterative reconstruction (iDose) and virtual monoenergetic imaging at 50 keV. Four radiologists independently and randomly reviewed the de-identified iDose and 50 keV images. Lesion detection, CRLM conspicuity, and CRLM diagnosis were compared between these images using a generalized estimating equation analysis. The reference standards used were histopathology and follow-up imaging findings.

**Results:**

The study included 797 focal liver lesions, including 463 CRLMs (median size, 18.1 mm [interquartile range, 10.9–37.7 mm]). Lesion detection was better with 50 keV images than with iDose images (45.0% [95% confidence interval [CI]: 39–50] vs 40.0% [95% CI: 34–46], *P* = 0.003). CRLM conspicuity was higher in the 50 keV images than in the iDose images (3.27 [95% CI: 3.09–3.46] vs 3.09 [95% CI: 2.90–3.28], *P* < 0.001). However, the specificity for diagnosing CRLM was lower with 50 keV images than with iDose images (94.5% [95% CI: 91.6–96.4] vs 96.0% [95% CI: 93.2–98.1], *P* = 0.022), whereas sensitivity did not differ significantly (77.6% [95% CI: 70.3–83.5] vs 76.9% [95% CI: 70.0–82.7], *P* = 0.736). Indeterminate lesions were more frequently noted in 50 keV images than in iDose images (13% [445/3188] vs 9% [313/3188], *P* = 0.005), and 56% (247/445) of the indeterminate lesions at 50 keV were not CRLMs.

**Conclusion:**

The 50 keV images obtained from DLSCT were better than the iDose images in terms of CRLM conspicuity and lesion detection. However, 50 keV images did not improve CRLM diagnosis but slightly increased the reporting of indeterminate focal liver lesions associated with CRLMs.

**Supplementary Information:**

The online version contains supplementary material available at 10.1007/s00261-024-04635-8.

## Introduction

Colorectal cancer (CRC) is a significant global health concern because it tends to metastasize, most often targeting the liver [1]. Detection of colorectal liver metastasis (CRLM) is important for staging and predicting the prognosis of patients with CRC [2]. Early detection of CRLM is crucial for identifying which patients would benefit from hepatic resection versus those for whom chemotherapy is more appropriate [3].

Contrast-enhanced CT is the standard imaging modality for CRC staging, and magnetic resonance imaging is recommended only for patients suspected of having CRLM to improve diagnostic accuracy [4]. Therefore, improving the depiction of CRLM on CT scans is important, and various strategies, including spectral CT, have been implemented [5]. Spectral CT enables the creation of virtual monoenergetic images (VMI) [6, 7]. At low-keV levels (below 70 keV), VMI enhances iodine contrast in the liver, thereby improving the contrast between the liver parenchyma and focal liver lesions [7, 8]. According to a recent systematic review, 61% of DECT studies have reported focal lesion detection. Although improved focal liver lesion detection has been consistently reported for hypervascular or hyperattenuating lesions [9], comparative results with conventional images on hypoattenuating lesions are inconsistent [10, 11].

Therefore, we aimed to assess the potential of VMI obtained from spectral CT in evaluating CRLM compared to conventional CT images.

## Materials and methods

The Institutional Review Board of Seoul National University Hospital (IRB No. H-1910-156-1073) approved this retrospective study and waived the requirement for informed consent.

### Patients

**Fig. 1:**
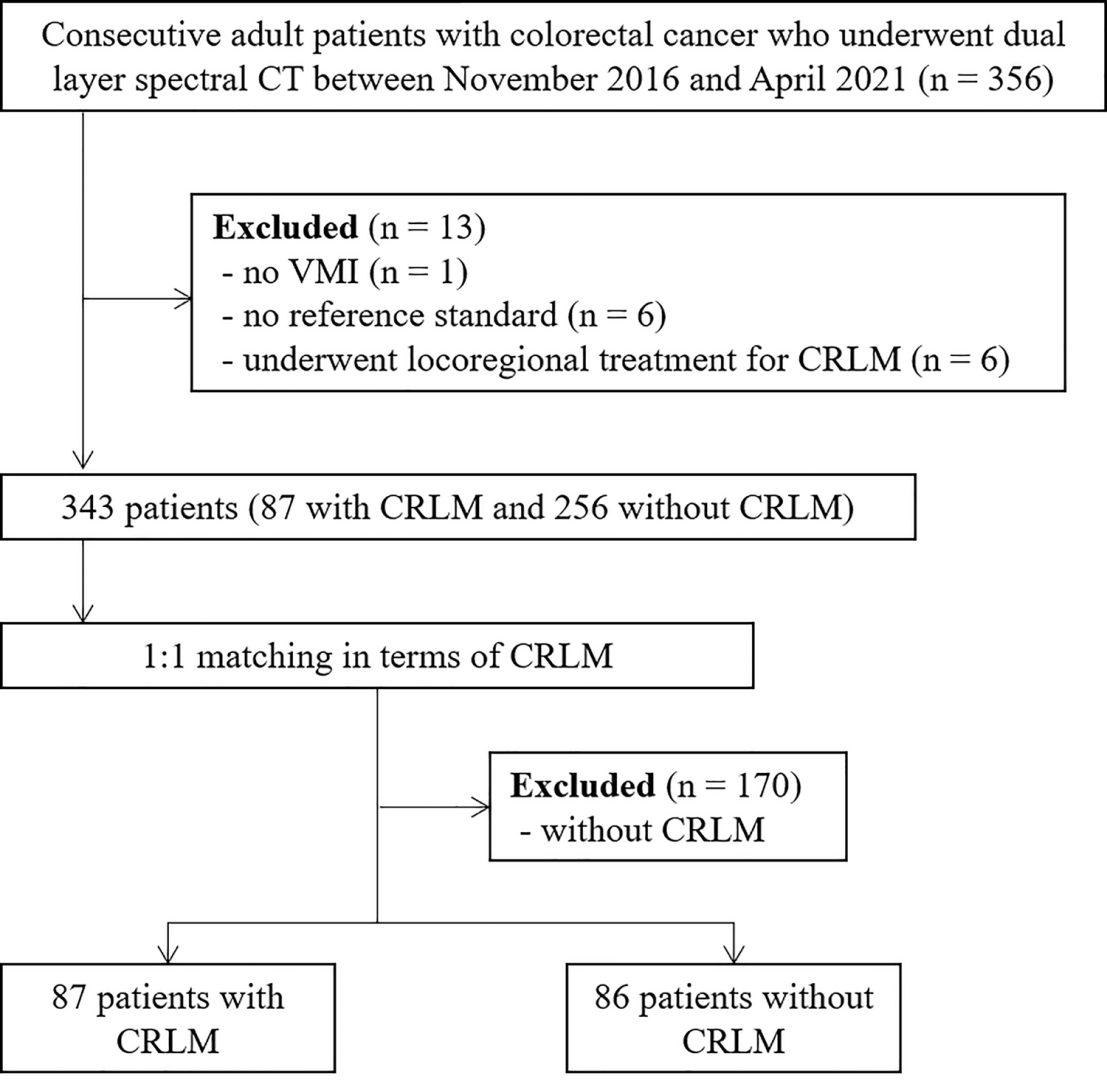
Study flow diagram. *VMI* virtual monoenergetic images, *CRLM* colorectal liver metastasis

From the electronic database of Seoul National University Hospital, we identified 356 consecutive adult patients with CRC who underwent dual-layer spectral CT (DLSCT) between November 2016 and April 2021. A study coordinator (J.S.B., a board-certified radiologist with 7 years of experience in abdominal imaging after fellowship) reviewed the medical records to identify eligible patients. After that, the following exclusion criteria were used: (a) absence of available VMI at 50 keV, (b) lack of a reference standard (see below), and (c) prior locoregional treatment for CRLM. After applying these criteria, 343 patients remained eligible (87 with CRLM and 256 without CRLM). Among them, we selected 173 patients with or without CRLMs through 1:1 matching based on body mass index (87 with CRLM and 86 without CRLM) (Fig. 1).

### Reference standards

We used a composite reference standard to determine the presence or absence of CRLM. Pathological diagnosis from surgery or biopsy served as a reference for patients with available pathology. For patients without pathology, follow-up CT or gadoxetic acid-enhanced MRI within 12 weeks of DLSCT was used as the reference standard to determine the number of focal liver lesions (FLLs). For FLL characterization, MRI and follow-up CT were used, and interval between DLSCT and follow-up CT/MRI was not restricted to be within 12 weeks. Hypermetabolic uptake on ^18^F-FDG-PET within 12 weeks of DLSCT was also used to diagnose CRLMs when available. Additional details of the imaging diagnosis of CRLM are provided in the Supplementary Material.

### Image acquisition and postprocessing

All examinations were performed using a DLSCT scanner (IQon; Philips Healthcare). The CT parameters used in this study included a rotation time of 0.33 s, peak voltage of 120 kVp, and tube current of 150–250 mAs. Following the acquisition of precontrast images, an intravenous contrast agent was administered by using a dosage of 1.6 mL/kg of 350 mgI/mL contrast media, with an infusion rate of 3–5 mL/s, followed by a saline flush of 20–40 mL using an automatic power injector (Table 1). In case of contrast media with concentration other than 350 mgI/mL due to hypersensitivity, the total amount of contrast media was adjusted by reducing or increasing the volume. Only portal venous phase (PVP) images were used in this study. PVP axial images were obtained using the bolus tracking technique with scan delays set at 55–70 s, initiated upon reaching a threshold enhancement of 150 HU in the distal thoracic aorta.

**Table 1:** Patient characteristics

Characteristics	Value
Sex (male: female)	106: 67 (61.3: 38.7)
Age, y	65 (58–74)
Height, cm	164 (157–168)
Weight, kg	60.7 (55.0–67.9)
Body mass index, kg/m^2^	22.7 (21.3–25.7)
Hepatic steatosison CT	6 (3.5)
*Number of CRLMs per patient*	
1	63 (36.4)
2	13 (7.5)
3	10 (5.8)
4	11 (6.4)
≥ 5	76 (43.9)
*Location of primary cancer*	
Colon	126 (72.8)
Rectum	47 (27.2)
*Mucinous*	
Non-mucinous	144 (83.2)
Mucinous	29 (16.8)
*Chronicity*^a^	
Synchronous	60 (69.0)
Metachronous	27 (31.0)
*Prior chemotherapy*	
Yes	147 (85.0)
No	26 (15.0)
*Reference standard*	
Pathology	16 (9.2)
Gadoxetic acid-enhanced MRI	6 (3.5)
Follow-up CT	151 (87.3)
*Radiation dose*	
CTDIvol (mGy)	25.9 (22.6–30.2)
Total DLP (mGy*cm)	1008.6 (858.1–1192.3)
CT contrast used	
Iohexol 350	146 (84.4)
Iobitridol 350	21 (12.1)
Others^b^	6 (3.5)
Contrast agent dose (ml)	97.1 ± 15.5 (60–130)

Values are presented as numbers (percentages), medians (ranges) or mean ± standard deviation (range)

*CRLM* colorectal liver metastasis, *CTDIvol* volume CT dose index, *DLP* dose–length product

^a^Evaluated only in 87 patients with CRLMs

^b^Ioversol 320 (n = 3), Iomeprol 400 (n = 2) and Iohexol 400 (n = 1)

Conventional images were reconstructed using a hybrid iterative reconstruction algorithm (iDose4, Philips Healthcare) with a standard soft tissue kernel. VMI images of 50 keV were reconstructed using a dedicated, hybrid iterative spectral reconstruction algorithm (Spectral, Kernel B, Philips Healthcare). Among the energy levels, 50 keV was chosen because it is considered an optimal compromise between image contrast, image quality, and lesion conspicuity [12, 13]. Both the iDose and 50 keV images were reconstructed using a section thickness of 3.0 mm and an interval of 2.0 mm.

### Qualitative image analysis

Four board-certified radiologists (J.H.K, S.W.K., S.P., and S.H., each with 2 years of experience in abdominal imaging after fellowship) independently reviewed the de-identified images in a random order. The reviewers were permitted to adjust the window width and level by using a picture archiving and communication system. A four-point scale was used to assess image noise, image contrast, and overall image quality, with higher scores indicating superior image quality (details in Supplementary Table 1). The reviewers recorded the location and size of the FLLs, excluding definite simple cysts and hemangiomas on CT. Lesion conspicuity during PVP was assessed on a 4-point scale: 1, no visualization (missed lesions); 2, barely visualized; 3, clear contrast with a partially blurry margin or modest contrast with a clear margin; and 4, clear contrast with a clear border [14].

Additionally, reviewers graded the detected FLLs for the probability of CRLMs using the following scale: 1, definitely benign; 2, probably benign; 3, indeterminate; 4, probably metastatic; and 5, definitely metastatic [15]. FLLs graded 4 or 5 were categorized as CRLMs. Image analyses were conducted independently for each image set, with a 4-week interval between evaluations, to minimize recall bias. Furthermore, the reviewers were asked if they would recommend an additional assessment of the FLL at their discretion. If they answered yes, they were requested to specify the preferred workup modality, such as gadoxetic acid-enhanced MRI.

### Quantitative image analysis

For quantitative analysis, a board-certified radiologist (J.S.B.) manually placed three circular regions of interest on the subcutaneous fat layer of the anterior abdominal wall, liver, and portal vein on three consecutive CT slices. The average Hounsfield unit (HU) value of the three regions of interest for each organ was used. Image noise was defined as the standard deviation of the HU value in the subcutaneous fat layer. Contrast-to-noise ratios (CNRs) of the portal vein were calculated as follows [16].

$$\text{CNR of portal vein}=\frac{{HU}_{portal vein} -{ HU}_{liver parenchyma}}{Image\, \, noise}$$

In addition, precontrast CT images were assessed to determine the presence of fatty liver, which has been reported to impact the evaluation of FLL [17]. The radiologist (J.S.B.) reviewed the CT images to identify patients with hepatic steatosis by using a previously established CT index based on the attenuation of the liver and spleen [18, 19]. If the attenuation difference between the liver and spleen was greater than − 9 (liver—spleen < − 9), it was considered indicative of hepatic steatosis.

### Statistical analysis

The independent t-test or Wilcoxon rank-sum test was performed for the comparison of continuous variables, and the chi-square test or the Fisher exact test for categorical variables, as appropriate. Interobserver agreement was assessed using Gwet’s AC2 [20, 21]. For per-lesion analysis, the generalized estimating equation (GEE) approach was used to estimate and compare image quality, lesion conspicuity, and lesion performance (detection, sensitivity, specificity, positive predictive value [PPV], negative predictive value [NPV], and accuracy) by using all the results from four radiologists. Identity-link GEE was used for lesion conspicuity, and log-link GEE was used for other metrics. For per-patient analysis, a multi-reader multi-case analysis was conducted for sensitivity, specificity, and accuracy, with logit-link GEE used for PPV and NPV. Lesion conspicuity was evaluated only for CRLMs, whereas lesion detection was assessed for FLLs. Subgroup analysis was performed based on the lesion size (≤ 10 mm or > 10 mm). Statistical analyses were performed using commercially available software packages: Medcalc (Medcalc Software), SAS version 9.4 (SAS Institute Inc.), and R for Windows version 4.2.3 (R package – MRMCaov). Statistical significance was set at *P* < 0.050.

## Results

### Patients and lesions

A total of 173 patients were included in the study (mean age, 65.5 ± 10.6 years; 106 male) with 463 CRLMs (median size, 18.1 mm [interquartile range, 10.9–37.7 mm]) (Table 1). Approximately 22.9% (106/463) of CRLMs were ≤ 10 mm in size. Image quality was assessed in all 173 patients, and lesion conspicuity was evaluated in 463 CRLMs in 85 patients. The reference standard included histopathology (n = 16) for 13 patients with CRLM and three patients without CRLM, gadoxetic acid-enhanced MRI (n = 6) for six patients with CRLM, and follow-up CT imaging for the remaining 151 patients.

### Image quality

**Fig. 2:**
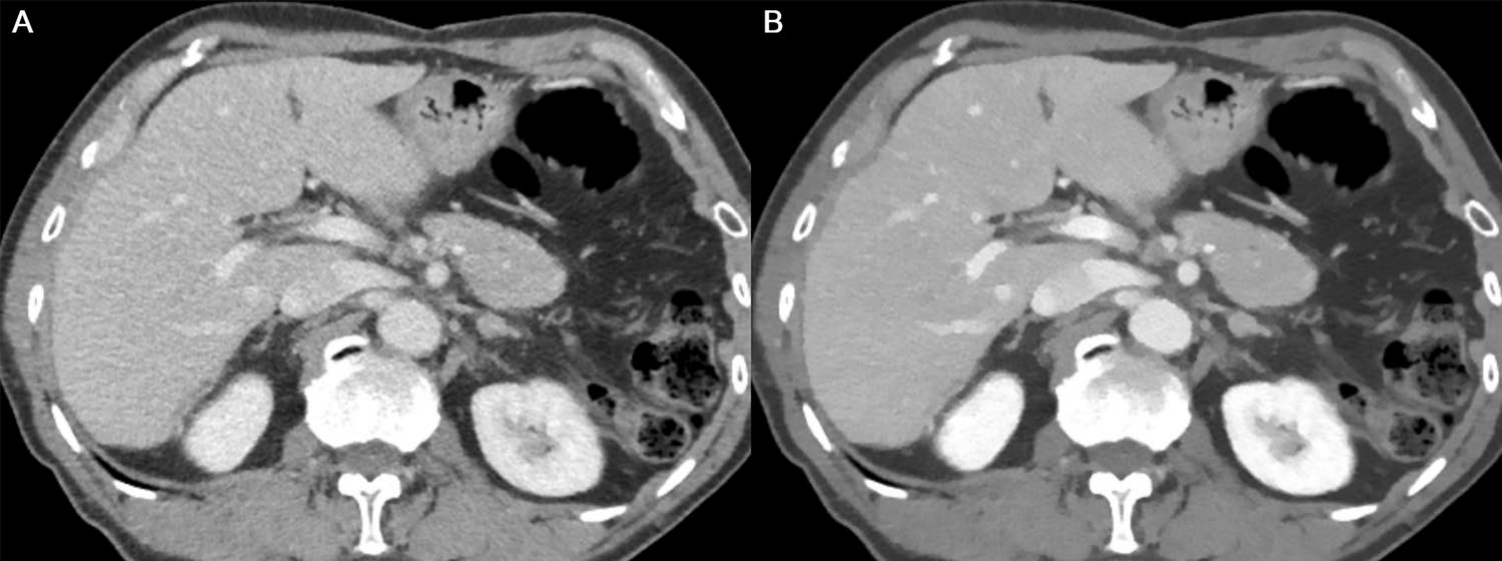
Comparison of image quality between iDose images and 50 keV images in a 71-year-old man. Compared with the iDose images (**A**), the 50 keV images (**B**) show less image noise and better image contrast

The 50 keV images showed significantly lower noise, higher contrast, and superior overall image quality than iDose images (3.73 vs 3.07, 3.98 vs 3.03, and 3.57 vs 3.05, respectively, *P* < 0.001 for all) (Table 2 and Fig. 2). The results from each reviewer are presented in Supplementary Table 2. Quantitative analysis revealed that image noise was not significantly different between iDose images and 50 keV images (6.37 [95% confidence interval [CI], 6.17–6.57] vs. 6.52 [95% CI, 6.29–6.75], *P* = 0.334), whereas CNR of portal vein was significantly higher with 50 keV images than with iDose images (28.87 [95% CI, 27.3–30.4] vs. 11.37 [95% CI, 10.8–12.0], *P* < 0.001).

**Table 2:** Comparison of image quality and lesion conspicuity between iDose images and 50 keV images

	iDose images	50 keV images	*P* value
*Image quality*			
Image noise	3.07 [3.03, 3.11]	3.73 [3.69, 3.77]	< 0.001
Image contrast	3.03 [2.99, 3.07]	3.98 [3.96, 3.99]	< 0.001
Overall image quality	3.05 [3.01, 3.10]	3.57 [3.53, 3.61]	< 0.001
*Lesion conspicuity*			
All CRLMs (n = 463)	3.09 [2.90, 3.28]	3.27 [3.09, 3.46]	< 0.001
CRLMs ≤ 10 mm (n = 106)	2.36 [2.09, 2.64]	2.71 [2.43, 2.99]	< 0.001
CRLMs > 10 mm (n = 357)	3.34 [3.17, 3.51]	3.47 [3.30, 3.64]	< 0.001

Values are estimated using two-sided 95% confidence intervals in parentheses

*CRLM* colorectal liver metastasis

The interobserver agreement showed AC2 values of 0.589 (95% CI: 0.557, 0.624) for image noise, 0.842 (95% CI: 0.812, 0.868) for image contrast, and 0.483 (95% CI: 0.448, 0.515) for overall image quality.

### CRLM conspicuity

**Fig. 3:**
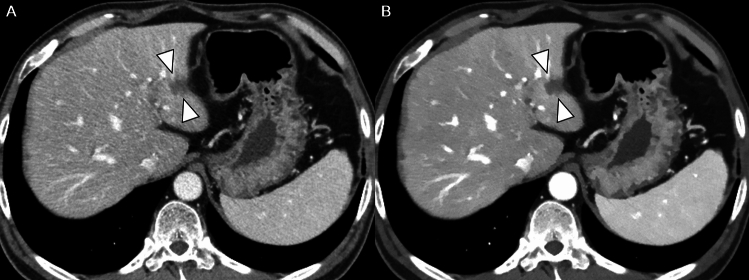
Comparison of lesion conspicuity between iDose images and 50 keV images. A 58-year-old man presented with a 15 mm-sized CRLM in the left lateral segment of the liver (arrowheads). Better conspicuity of CRLM is noted in 50 keV images (**B**) than in iDose images (**A**)

Among all CRLMs, lesion conspicuity was significantly higher in the 50 keV images than in the iDose images (3.27 [95% CI, 3.09–3.46] vs 3.09 [95% CI, 2.90–3.28], P < 0.001) (Table 2 and Fig. 3). This superiority was consistent across lesion sizes: 2.71 (95% CI, 2.43–2.99) vs 2.36 (95% CI, 2.09–2.64) for CRLMs ≤ 10 mm (*P* = 0.001), and 3.47 (95% CI, 3.30–3.64) vs 3.34 (95% CI, 3.17–3.51) for CRLMs > 10 mm (*P* < 0.001) (Table 2).

### Lesion detection

**Fig. 4:**
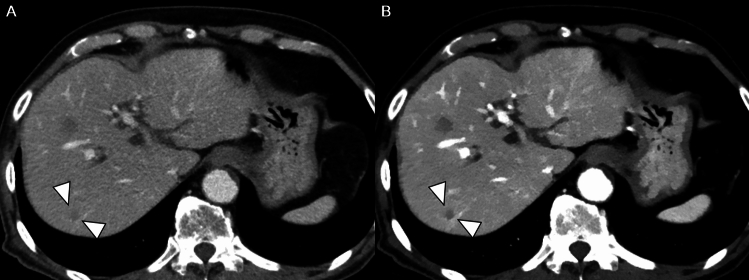
Comparison of lesion detectability between iDose images and 50 keV images in a 71-year-old man. Only one of the four readers detected a 6 mm-sized CRLM (arrowheads) in liver segment 7 on iDose images (**A**), whereas all four readers detected the lesion on the 50 keV images (**B**)

There were 797 FLLs, including 463 CRLMs and 334 non-CRLMs. Non-CRLM FLLs are clinically benign. In per-lesion analysis, lesion detection rates were higher with 50 keV images than with iDose images (45% [95% CI, 39–50%] vs 40% [95% CI, 34–46%], *P* = 0.003) (Table 3 and Fig. 4). In the subgroup analysis according to lesion size, lesion detection was better with 50 keV images than with iDose images (32% [95% CI, 26–37%] vs 23% [95% CI, 18–29%]), with a difference of 8.7% (95% CI, 3.4–14.0%; *P* = 0.002) in smaller lesions (≤ 10 mm). However, no significant difference was observed between the 50 keV and iDose images (71% [95% CI, 64–76%] vs 69% [95% CI, 62–76%]), with a difference of 1.4% (95% CI, − 0.6–3.4%; *P* = 0.173) in larger lesions (> 10 mm). On a per-patient basis, no significant difference was observed in the lesion detection rates between the 50 keV and iDose images (Table 3).

**Table 3:** Comparison of lesion detection between iDose images and 50 keV images

	iDose images	50 keV images	*P* value
*Per-lesion*			
All lesions (n = 797)	40 (1878/3188) [34, 46]	45 (2038/3188) [39, 50]	0.003
Lesions ≤ 10 mm (n = 371)	23 (447/1484) [18, 29]	32 (587/1484) [26, 37]	0.002
Lesions > 10 mm (n = 426)	69 (1431/1704) [62, 76]	71 (1451/1704) [64, 76]	0.173
*Per-patient*			
All patients (n = 173)	54 (374/692) [47, 61]	55 (383/692) [49, 62]	0.451
Patients with lesions ≤ 10 mm (n = 68)	15 (41/272) [10, 23]	17 (46/272) [11, 25]	0.522
Patients with lesions > 10 mm (n = 105)	79 (333/420) [72, 85]	80 (337/420) [73, 86]	0.658

Data represents combined results from four radiologists. Values are percentages (numerator/denominator) with two-sided 95% confidence intervals in parentheses

### CRLM diagnosis

On a per-lesion basis, sensitivity did not differ between 50 keV and iDose images (77.6% [95% CI, 70.3–83.5%] vs 76.9% [95% CI, 70.0–82.7%], *P* = 0.736) (Table 4). However, the specificity was lower with 50 keV images than with iDose images (94.5% [95% CI, 91.6–96.4%] vs 96.0% [95% CI, 93.2–97.7%], *P* = 0.022). PPV was also lower for 50 keV images than for iDose images (95.1% [95% CI, 91.6–97.2%] vs 96.4% [95% CI, 93.3–98.1%], *P* = 0.043). NPV and accuracy did not show significant differences between the 50 keV images and iDose images (*P* = 0.888 and 0.806, respectively). Subgroup analysis based on CRLM size (≤ 10 mm) showed no significant differences between the 50 keV and iDose images (Supplementary Table 3).

**Table 4:** Comparison of CRLM diagnosis between iDose images and 50 keV images

	iDose images	50 keV images	*P* value
*Per-lesion*			
Sensitivity (%)	76.9 (1425/1852) [70.0, 82.7]	77.6 (1437/1852) [70.3, 83.5]	0.736
Specificity (%)	96 (1283/1336) [93.2, 97.7]	94.5 (1262/1336) [91.6, 96.4]	0.022
PPV (%)	96.4 (1425/1478) [93.3, 98.1]	95.1 (1437/1511) [91.6, 97.2]	0.043
NPV (%)	75 (1283/1710) [67.5, 81.3]	75.3 (1262/1677) [67.3, 81.8]	0.888
Accuracy (%)	84.9 (2708/3188) [80.8, 88.3]	84.7 (2699/3188) [80.4, 88.1]	0.806
*Per-patient*			
Sensitivity (%)	76.2 (259/340) [65.8, 86.6]	79.1 (269/340) [69.1, 89.1]	0.592
Specificity (%)	95.7 (337/352) [90.3, 100]	94.3 (332/352) [86.1, 100.0]	0.555
PPV (%)	94.5 (259/274) [90.1, 97]	93.1 (269/289) [87.9, 96.1]	0.288
NPV (%)	80.6 (337/418) [72.8, 86.6]	82.4 (332/403) [74.7, 88.1]	0.236
Accuracy (%)	86.1 (596/692) [81.2, 91.1]	86.8 (601/692) [82.9, 90.8]	0.668

Data represents combined results from four radiologists. Values are estimates (numerator/denominator) with two-sided 95% confidence intervals in parentheses

*CRLM* colorectal liver metastasis, *PPV* positive predictive value, *NPV* negative predictive value

On a per-patient basis, sensitivity (79.1% [95% CI, 69.1–89.1%] vs 76.2% [95% CI, 65.8–86.6%]) and specificity (94.3% [95% CI, 86.1–100%] vs 95.7% [95% CI, 90.3–100%]) did not differ significantly between the 50 keV images and iDose images (*P* = 0.592 and 0.555, respectively) (Table 4). Similarly, the PPV, NPV, and accuracy did not show significant differences between the 50 keV images and iDose images (*P* = 0.288, 0.236, and 0.668, respectively). Subgroup analysis based on CRLM size (≤ 10 mm) did not reveal significant differences between 50 keV images and iDose images (*P* = 0.296–0.896, Supplementary Table 4).

### Indeterminate lesion

On a per-lesion basis, indeterminate lesions (CRLM probability score 3) were more frequently noted with 50 keV images than with iDose images (13% [95% CI, 11–15%] vs 9% [95% CI, 7–11%], *P* = 0.005) (Table 5). In the subgroup analysis, smaller lesions (≤ 10 mm) were more likely to be considered indeterminate on 50 keV images than on iDose images (18% [95% CI, 15–22%] vs 10% [95% CI, 8–13%], *P* < 0.001). For larger lesions (> 10 mm), no significant difference was observed between 50 keV images and iDose images (10% [95% CI, 8–13%] vs 10% [95% CI, 8–13%], *P* > 0.999). However, the actual proportion of CRLMs among the indeterminate lesions was significantly lower with 50 keV images than with iDose images (44% [95% CI, 33–57%] vs 60% [95% CI, 47–73%]), with a difference of 15.9% (95% CI, 4.3–27.5%; *P* = 0.007) (Supplementary Table 5).

**Table 5:** Comparison of indeterminate lesion distribution between iDose images and 50 keV images

	iDose images	50 keV images	*P* value
*Per-lesion*			
All lesions (n = 797)	9 (313/3188) [7, 11]	13 (445/3188) [11, 15]	0.005
Lesions ≤ 10 mm (n = 371)	10 (189/1484) [8, 13]	18 (321/1484) [15, 22]	< 0.001
Lesions > 10 mm (n = 426)	10 (124/1704) [7, 13]	10 (124/1704) [8, 13]	> 0.999
*Per-patient*			
All patients (n = 173)	10 (72/692) [8, 14]	11 (74/692) [8, 14]	0.854
Patients with lesions ≤ 10 mm (n = 68)	8 (22/272) [5, 13]	10 (28/272) [6, 17]	0.429
Patients with lesions > 10 mm (n = 105)	12 (50/420) [8, 16]	11 (46/420) [7, 16]	0.605

Data represents combined results from four radiologists. Values are percentages (numerator/denominator) with two-sided 95% confidence intervals in brackets

On a per-patient basis, the distribution of patients with indeterminate lesions but without probable or definite metastasis did not differ between the 50 keV images and iDose images (Table 5). Similarly, the actual proportion of CRLMs among the indeterminate lesions was not significantly different between the 50 keV images and iDose images (39% [95% CI, 25–56%] vs 44% [95% CI, 30–60%]), with a difference of 5.3% (95% CI, − 9.3 to 19.8%; *P* = 0.479) (Supplementary Table 5). No significant difference was observed in the recommendation for further workup of indeterminate lesions between 50 keV images and iDose images (29% [95% CI, 24–34%] vs 27% [95% CI, 23–31%],* P* > 0.999). All the reviewers recommended gadoxetic acid-enhanced MRI as the modality of choice for further evaluation.

## Discussion

In this study, we assessed the potential of 50 keV images compared to conventional iDose CT images for assessing CRLM. Regarding image quality, 50 keV images demonstrated significantly less noise, higher contrast, and better overall image quality than iDose images (3.73 vs 3.07, 3.98 vs 3.03, and 3.57 vs 3.05, Ps < 0.001 for all), consistent with previous studies [6, 8, 22, 23]. Regarding the CRLM evaluation, lesion conspicuity was higher with 50 keV images than with iDose images (3.27 vs 3.09, *P* < 0.001) for both CRLMs ≤ 10 mm and > 10 mm. Lesion detection was also better with 50 keV images than with iDose images (45.0% vs 40.0%, *P* = 0.003) on a per-lesion basis. However, the specificity for diagnosing CRLM was lower with 50 keV images than with iDose images (94.5% vs 96.0%, *P* = 0.022) on a per-lesion basis. Indeterminate lesions were more frequently noted with 50 keV images than with iDose images (13% vs 9%, *P* = 0.005) but were less likely to be confirmed as CRLMs with 50 keV images than with iDose images (44% vs 60%, *P* = 0.007).

Lesion detection is crucial in the initial assessment of CRLMs; in this regard, 50 keV images demonstrated superior performance compared with iDose images. This enhanced performance of 50 keV images was particularly evident in detecting small (≤ 10 mm) CRLMs, addressing the limitations of conventional CT in visualizing small FLLs. These results align with previous studies that have reported superior diagnostic performance of VMI compared to conventional imaging techniques [6, 8, 22, 23]. Detection of small CRLMs can facilitate prompt diagnosis and appropriate treatment. Therefore, 50 keV images may offer a greater diagnostic value than conventional images for evaluating patients with CRC. In addition, 50 keV images showed better lesion conspicuity than iDose images, which may enhance the radiologists’ confidence in detecting and characterizing CRLMs.

Despite the improved detection of CRLM using 50 keV images, our study did not demonstrate a corresponding improvement in diagnostic accuracy. Specifically, the specificity and PPV of 50 keV images were lower than those of iDose images without a concurrent increase in sensitivity. This observation may be attributed to the increased liver-to-lesion contrast characteristic of the 50 keV images. Despite the theoretical advantage of low monoenergetic images for enhancing the diagnosis of FLLs, previous studies have consistently reported negligible differences in the diagnosis of liver metastasis [15, 24]. This discrepancy may be related to the different types of errors encountered in the radiology readings. We hypothesized that while the high contrast of 50 keV images may help in detecting FLLs more effectively, it may not necessarily reduce classification errors [25]. This hypothesis is consistent with a recent study that reported inconsistencies between detection and classification errors [26]. Our hypothesis appears reasonable, especially considering the observed improved performance of 50 keV in detecting FLLs ≤ 10 mm, which is often challenging to characterize owing to their small size.

One notable observation was the higher frequency of indeterminate lesions for CRLM reported in 50 keV images, many of which were eventually determined not to be CRLM. It echoes a recent study reporting discrepancy between detection and PPV in small (< 6 mm) FLLs at 70 keV images [27]. Although we cannot provide a clear explanation for this observation, it may be related to alterations in the image texture at 50 keV and partial volume averaging artifacts caused by prominent surrounding parenchymal enhancement [28]. Although this did not result in significant differences at the per-patient level or in the frequency of recommending further evaluation, radiologist caution is needed. However, further studies are required to address this issue.

This study had a few limitations. First, it was a retrospective single-center study, which may have introduced a selection bias. Second, the reference standards rely on imaging examinations for most lesions, thereby limiting the accurate characterization of non-CRLM lesions. Third, we evaluated the VMI obtained from only a single type of DLSCT machine, which may restrict the generalizability of our results to VMI generated using other dual-energy CT machines from different manufacturers. Lastly, a small proportion of our patients (3.5%) who used contrast agent other than 350 mgI/mL. However, we managed to deliver similar total dose of iodine by adjusting contrast media volume in such cases and the impact would be minimal.

In conclusion, we found that 50 keV images significantly improved image quality, FLL detectability, and enhanced CRLM conspicuity compared to iDose images. These advantages highlight the relevance of 50 keV images in clinical practice. However, it is noteworthy that while 50 keV images did not improve CRLM diagnosis, they led to a slight increase in the reporting of indeterminate FLLs for CRLMs.

## Supplementary Information

Below is the link to the electronic supplementary material.

Supplementary file1 (DOCX 31 KB)

## Data Availability

No datasets were generated or analysed during the current study.
