# Supplementary material for: Evaluation of colorectal liver metastases using virtual monoenergetic images obtained from dual-layer spectral computed tomography
Source: Abdom Radiol (NY). 2024 Oct 15;50(4):1624–32. doi: 10.1007/s00261-024-04635-8 (PMC11946942; doi:10.1007/s00261-024-04635-8)
Supplement: Supplementary file 1 — Supplementary file1 (DOCX 31 KB) [file 261_2024_4635_MOESM1_ESM.docx]

**Supplementary material**

*Clinical diagnosis of CRLM*

For the lesions without pathological information, we used the imaging features on CT, MRI and/or ^18^F-FDG-PET to diagnose colorectal liver metastases (CRLMs) and relied on the results of imaging examinations such as gadoxetic acid-enhanced MRI or follow-up CT within 12 weeks of DLSCT to determine the number of focal liver lesions (FLLs). First, FLLs with targetoid imaging features, restricted diffusion, or hypermetabolic uptake on ^18^F-FDG-PET were thought to be CRLMs. Second, FLLs showed change in size on follow-up examinations in concordant with other FLLs with aforementioned CRLM features were also considered CRLMs. Newly detected FLLs on MRI or follow-up CT within 12 weeks of DLSCT were regarded as occult metastasis or missed FLLs on DLSCT. For FLL characterization, MRI and follow-up CT were used, and interval between DLSCT and follow-up CT/MRI was not restricted to be within 12 weeks. FLLs were regarded as benign if it showed characteristic imaging features on MRI or CT: peripheral nodular enhancement or hyperintensity on T2-weighted image for hemangioma, fat attenuation or signal drop on fat-suppressed images/in-opposed images for lipoma, or fluid-attenuation or hyperintensity on heavily T2-weighted image without enhancement for hepatic cysts (91 FLLs). FLLs without such characteristic features or too small to be accurately characterized were regarded as non-CRLM when these FLLs were stable for more than 6–12 months as patients with colorectal cancer are followed in every 3 to 6 months depending on their stage in our institution.

**Supplementary Table 1. Scoring scale for qualitative image analyses**

| Items | **Score** | **Scoring system** |
| --- | --- | --- |
| Image noise | 1–4 | Score 1, undiagnostic;  Score 2, diagnostic, but substantial noise;  Score 3, diagnostic, mild noise;  Score 4, no or minimal noise |
| Image contrast | 1–4 | Score 1, poor contrast (similar to precontrast CT)  Score 2, low contrast  Score 3, average contrast  Score 4, good/very strong contrast |
| Overall image quality | 1–4 | Score 1, undiagnostic  Score 2, diagnostic but significantly hampered image quality  Score 3, average  Score 4, better than average |

**Supplementary Table 2. Comparison of image quality between iDose images and 50keV images**

|  | **iDose images** | **50keV images** |
| --- | --- | --- |
|  |  |  |
| **Image noise** |  |  |
| Reader 1 | 3.00 [3.00, 4.00] | 4.00 [3.00, 4.00] |
| Reader 2 | 3.00 [3.00, 3.00] | 4.00 [4.00, 4.00] |
| Reader 3 | 3.00 [3.00, 3.00] | 4.00 [3.00, 4.00] |
| Reader 4 | 3.00 [3.00, 3.00] | 4.00 [3.00, 4.00] |
| **Image contrast** |  |  |
| Reader 1 | 3.00 [3.00, 3.00] | 4.00 [4.00, 4.00] |
| Reader 2 | 3.00 [3.00, 3.00] | 4.00 [4.00, 4.00] |
| Reader 3 | 3.00 [3.00, 3.00] | 4.00 [4.00, 4.00] |
| Reader 4 | 3.00 [3.00, 3.00] | 4.00 [4.00, 4.00] |
| **Overall image quality** |  |  |
| Reader 1 | 3.00 [3.00, 3.00] | 4.00 [4.00, 4.00] |
| Reader 2 | 3.00 [3.00, 3.00] | 3.00 [3.00, 4.00] |
| Reader 3 | 3.00 [3.00, 4.00] | 4.00 [3.00, 4.00] |
| Reader 4 | 3.00 [3.00, 3.00] | 3.00 [3.00, 4.00] |

Values are medians with interquartile ranges in brackets.

**Supplementary Table 3. Comparison of CRLM diagnosis between iDose images and 50keV images: subgroup analysis on a per-lesion basis**

|  | **iDose images** | **50keV images** | ***P* value** |
| --- | --- | --- | --- |
|  |  |  |  |
| **Sensitivity** |  |  |  |
| Lesion size ≤ 10 mm | 0.420 (178/424) [0.307, 0.542] | 0.408 (173/424) [0.317, 0.505] | 0.851 |
| Lesion size > 10 mm | 0.873 (1247/1428) [0.820, 0.912] | 0.885 (1264/1428) [0.839, 0.919] | 0.293 |
| **Specificity** |  |  |  |
| Lesion size ≤ 10 mm | 0.978 (1037/1060) [0.964, 0.987] | 0.967 (1025/1060) [0.950, 0.979] | 0.083 |
| Lesion size > 10 mm | 0.891 (246/276) [0.787, 0.948] | 0.859 (237/276) [0.762, 0.920] | 0.095 |
| **PPV** |  |  |  |
| Lesion size ≤ 10 mm | 0.886 (178/201) [0.790, 0.941] | 0.832 (173/208) [0.723, 0.904] | 0.125 |
| Lesion size > 10 mm | 0.977 (1247/1277) [0.938, 0.991] | 0.970 (1264/1303) [0.931, 0.987] | 0.138 |
| **NPV** |  |  |  |
| Lesion size ≤ 10 mm | 0.808 (1037/1283) [0.736, 0.864] | 0.803 (1025/1276) [0.721, 0.866] | 0.770 |
| Lesion size > 10 mm | 0.576 (246/427) [0.450, 0.693] | 0.591 (237/401) [0.469, 0.703] | 0.504 |
| **Accuracy** |  |  |  |
| Lesion size ≤ 10 mm | 0.819 (1215/1484) [0.761, 0.865] | 0.807 (1198/1484) [0.744, 0.858] | 0.537 |
| Lesion size > 10 mm | 0.876 (1493/1704) [0.831, 0.910] | 0.881 (1501/1704) [0.840, 0.912] | 0.633 |

Values are estimates (numerator/denominator) with two-sided 95% confidence intervals in brackets. CRLM = colorectal liver metastasis, PPV = positive predictive value, NPV = negative predictive value.

**Supplementary Table 4. Comparison of CRLM diagnosis between iDose images and 50keV images: subgroup analysis on a per-patient basis**

|  | **iDose images** | **50keV images** | ***P* value** |
| --- | --- | --- | --- |
|  |  |  |  |
| **Sensitivity** |  |  |  |
| Lesion size ≤ 10 mm | 0.167 (4/24) [<0.001, 0.389] | 0.250 (6/24) [<0.001, 0.586] | 0.536 |
| Lesion size > 10 mm | 0.807 (255/316) [0.710, 0.904] | 0.832 (263/316) [0.733, 0.932] | 0.641 |
| **Specificity** |  |  |  |
| Lesion size ≤ 10 mm | 0.988 (245/248) [0.975, 1.000] | 0.984 (244/248) [0.933, 1.000] | 0.810 |
| Lesion size > 10 mm | 0.885 (92/104) [0.714, 1.000] | 0.846 (88/104) [0.681, 1.000] | 0.423 |
| **PPV** |  |  |  |
| Lesion size ≤ 10 mm | 0.571 (4/7) [0.182, 0.889] | 0.600 (6/10) [0.205, 0.897] | 0.896 |
| Lesion size > 10 mm | 0.955 (255/267) [0.911, 0.978] | 0.943 (263/279) [0.890, 0.971] | 0.296 |
| **NPV** |  |  |  |
| Lesion size ≤ 10 mm | 0.925 (245/265) [0.837, 0.967] | 0.931 (244/262) [0.842, 0.972] | 0.579 |
| Lesion size > 10 mm | 0.601 (92/153) [0.456, 0.731] | 0.624 (88/141) [0.475, 0.753] | 0.426 |
| **Accuracy** |  |  |  |
| Lesion size ≤ 10 mm | 0.915 (249/272) [0.858, 0.973] | 0.919 (250/272) [0.857, 0.981] | 0.814 |
| Lesion size > 10 mm | 0.826 (347/420) [0.754, 0.899] | 0.836 (351/420) [0.776, 0.896] | 0.756 |

Values are estimates (numerator/denominator) with two-sided 95% confidence intervals in brackets. CRLM = colorectal liver metastasis, PPV = positive predictive value, NPV = negative predictive value.

**Supplementary Table 5. Comparison of the proportions of CRLMs among the indeterminate lesions between iDose images and 50keV images**

|  | **iDose images** | **50keV images** | ***P* value** |
| --- | --- | --- | --- |
|  |  |  |  |
| **Per-lesion** |  |  |  |
| All lesions | 60 (189/313) [47, 73] | 44 (198/445) [33, 57] | 0.007 |
| Lesions ≤ 10 mm | 57 (108/189) [43, 70] | 41 (130/321) [27, 55] | 0.013 |
| Lesions > 10 mm | 65 (81/124) [40, 84] | 55 (68/124) [36, 72] | 0.122 |
| **Per-patient** |  |  |  |
| All patients | 44 (32/72) [30, 60] | 39 (29/74) [25, 56] | 0.479 |
| Patients with lesions ≤ 10 mm | 45 (10/22) [20, 74] | 21 (6/28) [6, 55] | 0.070 |
| Patients with lesions > 10 mm | 44 (22/50) [27, 63] | 50 (23/46) [30, 70] | 0.468 |

Values are percentages (numerator/denominator) with two-sided 95% confidence intervals in brackets.
